# Supplementary material for: Dissecting multi drug resistance in head and neck cancer cells using multicellular tumor spheroids
Source: Sci Rep. 2019 Dec 27;9:20066. doi: 10.1038/s41598-019-56273-6 (PMC6934860; doi:10.1038/s41598-019-56273-6)
Supplement: Supplementary file 1 — Dissecting multi drug resistance in head and neck cancer cells using multicellular tumor spheroids [file 41598_2019_56273_MOESM1_ESM.docx]

**Dissecting multi drug resistance in head and neck cancer cells using multicellular tumor spheroids**

Mohammad Azharuddin^1,#^, Karin Roberg^1,2,*^, Ashis Kumar Dhara^3^, Mayur Vilas Jain^4^, Padraig Darcy^5^, Jorma Hinkula^1^, Nigel K H Slater^6^, Hirak K Patra^1,6, #,*^

^1^Department of Clinical and Experimental Medicine (IKE), Linkoping University, Linkoping, Sweden, ^2^Department of Otorhinolaryngology in Linköping, Anaesthetics, Operations and Specialty Surgery Center, Region Östergötland, Sweden, ^3^Department of Electrical Engineering, National Institute of Technology Durgapur, India, ^4^Division of Molecular Medicine and Gene Therapy, Lund University, Lund, Sweden, ^5^Department of Medical and Health Sciences (IMH), Division of Drug Research (LÄFO), Linköping University, Linköping, Sweden, ^6^Department of Chemical Engineering and Biotechnology, University of Cambridge, Cambridge, UK

*Correspondence: hp401@cam.ac.uk, [karin.roberg@liu.se](mailto:karin.roberg@liu.se) (H.K. Patra and Karin Roberg)

#Contributed equally


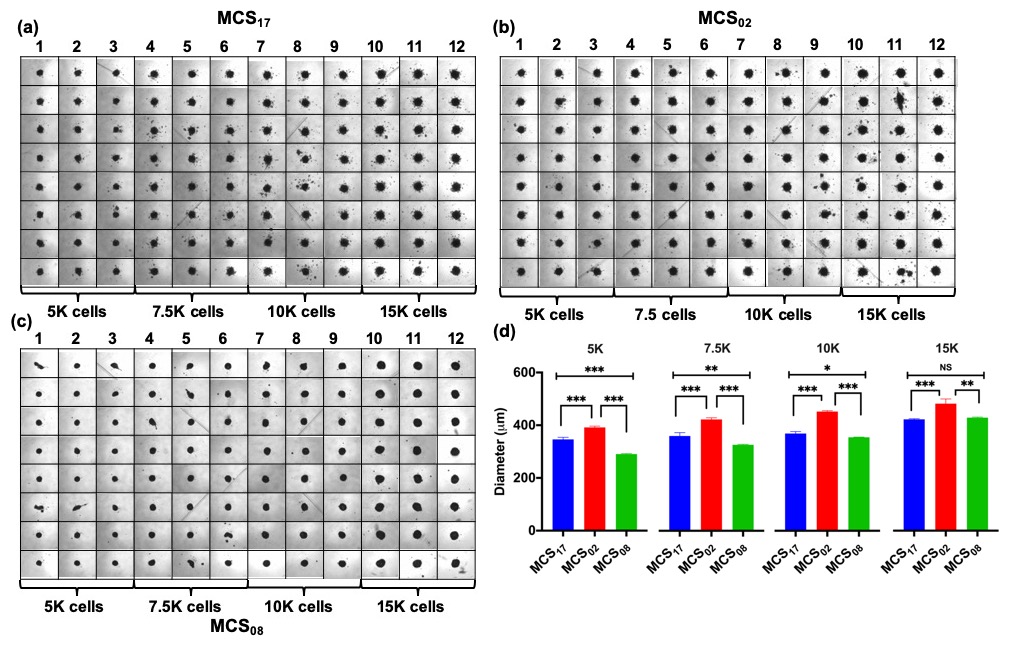


***Figure S1:*** *(a) – (c) Bright field images of MCSs (MCS_17_, MCS_02_, MCS_08_) for the three cell lines seeded at different cell density in ultra-low attachment plates acquired at 5x magnification. Cells were seeded at 5K, 7.5K, 10K, and 15K cells/ well and the corresponding diameter of the spheroids (b). The data are shown as a mean of ± SD, ***p<0.001, **p=0.001, *p=0.019. The images were acquired after 72 hours of spheroid formation.*
